# Cultural adaptation, perceived incentives, and job satisfaction of expatriate faculty: an empirical study of China and Kazakhstan

**DOI:** 10.3389/fpsyg.2026.1781942

**Published:** 2026-02-17

**Authors:** Chunling Wang, Abay K. Duisenbayev, Zhan Zhou

**Affiliations:** 1 Faculty of Philosophy and Political Science, Al-Farabi Kazakh National University, Almaty, Kazakhstan; 2 Continuing Education College, Chang'an University, Xi'an, China; 3 The Department of Pedagogy and Educational Management, Al-Farabi Kazakh National University, Almaty, Kazakhstan

**Keywords:** China-Kazakhstan educational cooperation, cultural adaptation, expatriate faculty, job satisfaction, perceived incentives

## Abstract

**Introduction:**

Within the context of the “Belt and Road” Initiative, faculty mobility between China and Kazakhstan has increased significantly. This study investigates the complex relationships between cultural adaptation, perceived incentives, and job satisfaction among expatriate faculty. It specifically addresses the theoretical gap in “South–South mobility” by examining how adaptation and organizational rewards interact to influence professional well-being across different national groups.

**Methods:**

Utilizing a quantitative survey design, data were collected from 550 expatriate faculty members working in universities in China and Kazakhstan. Analytical techniques included hierarchical regression, mediation and moderation analysis using the PROCESS macro, and two-way ANOVA to explore the impact of academic rank and tenure on satisfaction levels.

**Results:**

The findings reveal that: (1) Cultural adaptation and perceived incentives are both significant positive predictors of job satisfaction; (2) Perceived incentives partially mediate the relationship between cultural adaptation and job satisfaction, indicating that better adaptation is associated with a higher capacity to recognize and value organizational support; (3) Nationality moderates the link between incentives and satisfaction, with Kazakhstani faculty showing significantly higher sensitivity to extrinsic incentives than Chinese faculty; (4) A “satisfaction dip” exists for faculty in their first to third year of service and for those at the lecturer rank, who reported the lowest overall satisfaction scores.

**Discussion:**

The study extends acculturation theory by highlighting adaptation as a cognitive resource-acquisition capability rather than just an affective state. It demonstrates that the effectiveness of incentive structures is culturally contingent and follows different psychological contract orientations. Practically, these results suggest that universities should implement differentiated, full-cycle support systems and precision incentive strategies, specifically targeting early-career lecturers to mitigate turnover risks and enhance international academic cooperation.

## 1 Introduction

### 1.1 Research background and practical urgency

Under the dual drivers of economic globalization and the internationalization of higher education, the cross-border mobility of faculty has become a key feature of global higher education development (Shen et al., 2022). This trend is part of a broader transformation of “global work” in a rapidly changing world, presenting new implications for both organizations and individuals (Lazarova et al., 2023). Particularly within the context of the “Belt and Road” Initiative (BRI), educational cooperation between China and Central Asian countries, especially Kazakhstan, has entered a new historical phase. From the establishment of Confucius Institutes to the construction of Luban Workshops and the rise of joint research laboratories, a large number of Chinese faculty have been dispatched to teach in Kazakhstan, while a considerable number of Kazakhstani academics have been employed by Chinese universities.

Teaching abroad is not merely a geographical relocation but a profound psychological and professional challenge, often involving what Choi (2025) terms “toxic transitions” if not managed properly. Expatriate faculty face not only “Culture Shock” from language barriers and different living habits but also a host of other motivations and challenges unique to academic expatriates (Yao and Yang, 2024). They must adapt to foreign academic systems, evaluation criteria, and compensation structures. According to the existing literature, expatriates commonly experience identity reconstruction, professional role transition, and a re-evaluation of incentive mechanisms during their cultural adaptation process. These factors collectively influence their job satisfaction and work performance, and unchecked maladjustment can even pose significant workplace health risks (Choi, 2025).

Existing research has largely focused on expatriate managers in multinational corporations, with a notable lack of empirical studies on academic expatriates. This theoretical void is particularly glaring and significant in the context of “South–South Mobility”—the flow of talent between developing nations, of which the China-Kazakhstan faculty exchange is a prime example. Unlike the extensively studied “North–South” expatriates (e.g., a Western professor in a developing country), academics in a South–South context often lack the buffer of significant economic advantages or a sense of cultural superiority. This crucial distinction suggests that their psychological dynamics of adaptation, their response to organizational incentives, and their overall professional experience may follow a fundamentally different logic. As knowledge-intensive workers, their job satisfaction is not just a theoretical puzzle but a practical imperative, as it directly impacts teaching quality, research output, and ultimately, the sustainability and in-depth development of China-Kazakhstan educational cooperation.

### 1.2 Problem statement and research questions

In a transnational context, what determines the retention and satisfaction of an expatriate faculty member? While relevant literature exists, prior studies have not sufficiently clarified how adaptation and incentives interact within the specific context of South–South mobility. A key theoretical tension lies in whether Western-centric models of motivation apply equally to faculty moving between developing nations. This study addresses this gap by contextualizing Psychological Contract Theory, examining whether the contribution represents a theory refinement for this specific demographic. To ensure conceptual clarity throughout this study, we standardize the terminology as “Cultural Adaptation” to refer to the broader process of cross-cultural adjustment, and “Perceived Incentives” to denote the individual’s subjective evaluation of organizational rewards.

Specifically, this study focuses on the following three core research questions:

*RQ1* (mechanism question): How does cultural adaptation relate to job satisfaction through the mediating variable of perceived incentives? In other words, is “adapting well” linked closely to “more likely to perceive the institution’s goodwill,” thus becoming “more satisfied”?

*RQ2* (contextual question): Does nationality moderate the association of perceived incentives on job satisfaction? Do Chinese and Kazakhstani faculty exhibit significantly different response patterns to incentive measures due to their distinct cultural backgrounds, institutional environments, and types of psychological contracts? We treat findings related to nationality as tentative explorations of potential cultural differences.

*RQ3* (group question): What are the characteristic factors influencing the satisfaction of faculty groups with different academic ranks and lengths of service? Which groups represent “vulnerable populations” in expatriate faculty management that require targeted policy attention?

### 1.3 Significance of the study

*Theoretical significance*: This research makes several contributions to theory by bridging cross-cultural psychology and organizational behavior within the under-researched context of South–South mobility.

First, this study proposes and empirically tests an integrated “Adaptation-Incentive-Satisfaction” framework. By moving beyond separate analyses of cultural adaptation and job satisfaction, we investigate the mediating role of perceived incentives, thus illuminating a critical psychological pathway: effective adaptation is associated with an individual’s capacity to recognize and value organizational resources, which in turn is linked to satisfaction. This shifts the understanding of adaptation from a purely affective outcome to a cognitive resource-acquisition capability.

Second, and more importantly, this study contextualizes Psychological Contract Theory within the unique dynamics of South–South mobility. We hypothesize that the distinct institutional environments and motivations inherent in this context lead to different psychological contract orientations between national groups. Specifically, we will test whether Kazakhstani faculty in China tend towards a more transactional contract (emphasizing tangible, short-term exchanges), while Chinese faculty in Kazakhstan lean towards a relational contract (valuing long-term security and institutional mission). By examining how nationality moderates the impact of incentives on satisfaction, this research provides novel empirical evidence for the cultural contingency of psychological contracts and extends its applicability beyond traditional Western-centric, North–South expatriation models. This offers a nuanced theoretical lens for understanding why motivational logic may differ fundamentally in the Global South.

*Practical significance*: By precisely identifying the motivational pain points and adaptation barriers for faculty of different nationalities and experience levels, this study aims to provide data-driven support for Chinese and Kazakhstani universities to formulate more targeted talent recruitment policies, compensation systems, and support measures. The findings can be directly applied to: (1) optimize recruitment and selection standards for expatriate faculty (e.g., by adding cultural adaptation assessments); (2) design differentiated incentive packages (targeting the cultural sensitivities of different nationalities); (3) establish early-warning systems (to identify high-risk turnover groups); and (4) enhance cross-cultural training programs (to mitigate the negative impacts of culture shock).

## 2 Literature review and theoretical framework

### 2.1 Theoretical foundation

#### 2.1.1 The evolution of acculturation theory

Acculturation, a core concept in cross-cultural psychology, has evolved from a unidimensional to a multidimensional and from a static to a dynamic theoretical construct. Berry’s (1997) classic fourfold model (integration, assimilation, separation, marginalization) established the foundational framework for the field, emphasizing an individual’s choice of “dual identity” strategies regarding their heritage culture and the host culture.

In the 21st century, the ABC model (Affective-Behavioral-Cognitive Adaptation) proposed by Ward and Geeraert (2016) further refined acculturation into affective (psychological well-being, sense of belonging), behavioral (language proficiency, social skills), and cognitive (cultural intelligence, identity) dimensions. This multidimensional perspective better aligns with the complex experience of transnational professionals—an expatriate faculty member might be highly adapted behaviorally (e.g., fluent in the local language) yet still feel emotionally alienated, feeling, as Aydogan et al. (2025) vividly put it, “like a 6-year-old dropped on Mars without parents.” The cognitive dimension of this model, which includes cultural intelligence, is particularly salient for academics, as it involves the capability to function effectively in culturally diverse settings (Ang and Van Dyne, 2008). More recently, Ecological Systems Theory has further underscored the role of environmental factors. A meta-analysis by Bierwiaczonek and Waldzus (2016) indicated that organizational support (e.g., mentorship programs, cultural training) and community acceptance can sometimes have a greater impact on cultural adaptation than individual personality traits. This provides a theoretical rationale for incorporating “perceived incentives” (a form of organizational support) into our model.

#### 2.1.2 New developments in organizational incentive theory

The adjustment process of expatriates shares many similarities with that of organizational newcomers, requiring them to learn new roles and navigate unfamiliar social contexts (Dimitrova et al., 2023). Within this process, incentive theories offer crucial insights. Self-Determination Theory (SDT) offers a sophisticated framework for understanding the motivational needs of university faculty. Ryan and Deci (2000) identified three basic psychological needs: Autonomy, Competence, and Relatedness. For expatriate faculty, if the host university can satisfy these three needs—for instance, by granting academic freedom, providing professional development opportunities, and fostering an inclusive interpersonal atmosphere—their intrinsic motivation and satisfaction will significantly increase.

Psychological Contract Theory has gained prominence in expatriate research. Rousseau (1995) distinguished between a Transactional Contract (emphasizing short-term economic exchange) and a Relational Contract (emphasizing long-term emotional commitment). The violation of these contracts can lead to severe negative outcomes, such as displaced aggression, underscoring the importance of emotional regulation for expatriates (Schuster et al., 2022). This study posits that Chinese and Kazakhstani faculty may tend toward different types of psychological contracts: Kazakhstani faculty in China may lean more towards a transactional contract, while Chinese faculty in Kazakhstan may carry more relational contract expectations.

#### 2.1.3 Contextual theory in cross-cultural management: the uniqueness of south–south mobility

Traditional expatriate research has predominantly focused on “North–South” mobility (e.g., multinational corporations sending Western managers to Asia, Africa, or Latin America) or “South–North” mobility (e.g., talent from developing countries migrating to developed nations). However, south–south mobility—the flow of talent between developing countries—exhibits distinctly different characteristics.

Tung and Verbeke (2010) pointed out that South–South expatriates face a “double disadvantage”: they lack the high salary attraction of developed countries and the policy protections of their home country; they must overcome cultural differences while struggling to gain full acceptance in the host country. The exchange of faculty between China and Kazakhstan is a prime example of this model, fitting into the broader, yet under-researched, landscape of Global South research collaboration (Gueye et al., 2022). Furthermore, research suggests that the culture shock experienced by South–South expatriates is often underestimated. This is partly because both the home and host countries belong to the “developing world,” a context that can be associated with a form of “stigma” where the mobility itself is devalued (Xu et al., 2024). This leads organizations and individuals to mistakenly assume that “the differences won’t be that large,” thereby neglecting necessary cross-cultural training.

### 2.2 Cultural adaptation and job satisfaction

Cultural adaptation refers to the psychological, behavioral, and cognitive adjustments an individual makes when encountering a different cultural environment. A review by Ward (2001) noted that poor cultural adaptation often leads to role ambiguity, burnout, and turnover intention. Indeed, organizational support is a key factor perceived by expatriate academics that directly relates to their job satisfaction (Duffy, 2023).

In the context of higher education, the challenges of cultural adaptation are particularly salient. The university work environment is highly dependent on communication skills, interpersonal networking, and an understanding of unwritten rules (e.g., criteria for promotion, logic of research funding allocation). For example, a study by Schartner et al. (2023) on internationally mobile academics working in Thailand confirmed that intercultural adjustment is a critical factor for their professional and personal well-being. Their findings indicate that difficulties in adaptation negatively predict job satisfaction and are associated with higher turnover intentions. This is also linked to internal psychological resources, as recent work suggests that psychological capital can act as a catalyst to unlock job satisfaction and embeddedness among international faculty (Dev et al., 2025).

However, existing research has two main limitations: first, most studies use a cross-sectional design, which can only establish correlation, not causation; second, mediating mechanisms are often overlooked, with few studies exploring how cultural adaptation affects satisfaction or long-term career success after the assignment (Mello et al., 2023).

Based on the above analysis, this study proposes:

*Hypothesis H1*: The cultural adaptation level of expatriate faculty positively predicts their job satisfaction.

### 2.3 The mediating role of perceived incentives

Perceived Incentives refer to an individual’s subjective evaluation of the various rewards offered by an organization. In this study, to avoid conceptual ambiguity, we define “Perceived Incentives” as a composite construct reflecting the total value of organizational resources as perceived by the employee, encompassing both material elements (compensation, funding) and psychological elements (autonomy, recognition). While these originate from different traditions, they collectively form the “total reward” experience for the expatriate. According to Vroom’s (1964) Expectancy Theory, objective incentive measures can only be converted into psychological motivation when they are “perceived” and valued by the individual.

This study argues that cultural adaptation is a key antecedent of perceived incentives. This hypothesis is based on the following logic:

*Cognitive schemas*: Well-adapted faculty possess more accurate “cultural scripts,” enabling them to better interpret organizational signals. For example, a fully adapted faculty member can understand the promotion standards of the host university and interpret research funding support as a benevolent gesture from the organization to help them meet those standards.

*Information access*: Cultural adaptation includes language proficiency and the development of social networks, which are crucial for accessing information about organizational incentives. As Ma and Black (2025) argue, the development of social connections, or “ties,” is fundamental for expatriates. Without effective social networks, their lives can become “fragile,” limiting their ability to understand and access organizational support systems, thus lowering their perception of available incentives.

*Affective transfer*: According to Affective Events Theory (Weiss and Cropanzano, 1996), faculty mired in culture shock are often in a negative emotional state, which can color their evaluations of organizational behaviors.

When faculty perceive a high level of incentives, according to Social Exchange Theory, they feel an obligation to reciprocate, which in turn leads to a greater sense of achievement and satisfaction.

Therefore, this study proposes:

*Hypothesis H2*: Perceived incentives positively predict job satisfaction.

*Hypothesis H3*: Perceived incentives mediate the relationship between cultural adaptation and job satisfaction.

### 2.4 The moderating role of nationality: a China-Kazakhstan comparative perspective

Although China and Kazakhstan are geographically close, significant differences exist in their higher education governance models, academic cultures, and social contracts. These differences may lead faculty from the two countries to exhibit different patterns in the “incentive-satisfaction” mechanism.

#### 2.4.1 Differences in institutional logic

The different governance models of higher education in China and Kazakhstan lead to different psychological expectations among faculty. The public-sector nature of Chinese universities cultivates a sense of job security (the ‘iron rice bowl’ mentality), whereas market-oriented reforms in Kazakhstan have reinforced an ‘efficiency orientation’ among faculty.

#### 2.4.2 Differences in psychological contract types

Research into the employment relationships of Chinese expatriates suggests a tendency towards forming complex, multi-foci psychological contracts. For instance, Wang et al. (2023) found that Chinese expatriates’ psychological contracts are often relational, involving expectations of long-term security and organizational support that go beyond simple economic exchange. This contrasts with what might be expected from host-country employees, who may be more inclined towards transactional contracts emphasizing clear, material “payment for services rendered.”

This study proposes following theoretical expectations:

*Kazakhstani faculty in China*: May tend towards a transactional contract, focusing on “what tangible benefits can this job provide me?” Therefore, the provision of incentives (especially material ones) will have a substantial impact on their satisfaction.

*Chinese faculty in Kazakhstan*: May carry more relational contract expectations, viewing themselves as “cultural ambassadors.” Even if material conditions are slightly inferior, their satisfaction may remain at an acceptable level due to a sense of mission or the expectation of a secure position upon returning home.

Therefore, this study proposes:

*Hypothesis H4*: Nationality moderates the relationship between perceived incentives and job satisfaction.

## 3 Research methodology

### 3.1 Participants and procedure

This study selected Kazakhstani faculty employed by Chinese universities and Chinese faculty employed by Kazakhstani universities as its survey population. Using a snowball sampling method, bilingual questionnaires (Chinese/Russian) were distributed via email and WeChat/WhatsApp work groups. Data was collected from January to March 2024. A total of 600 questionnaires were returned. To ensure data quality, an attention check question (“Please select the ‘disagree’ option”) was included. After removing 50 invalid questionnaires that failed the attention check or showed patterned responses, 550 valid samples were obtained (an effective rate of 91.7%). The demographic characteristics of the sample are shown in Table 1.

**Table 1:** Sample demographic characteristics (*N* = 550).

Characteristic	Category	Frequency	Percentage (%)
Nationality	Chinese	275	50.0
	Kazakhstani	275	50.0
Gender	Male	269	48.9
	Female	281	51.1
Age	<30	82	14.9
	30–40	231	42.0
	41–50	178	32.4
	>50	59	10.7
Education	Bachelor’s	83	15.1
	Master’s	230	41.8
	PhD	237	43.1
Academic rank	Lecturer/assistant	198	36.0
	Associate professor	231	42.0
	Professor	98	17.8
	Other	23	4.2
Tenure (years)	<1	104	18.9
	1–3	187	34.0
	3–5	143	26.0
	>5	116	21.1

### 3.2 Measurement instruments

All scales used a 5-point Likert scale (1 = Strongly Disagree, 5 = Strongly Agree). The questionnaire included the following three core scales (see Supplementary material for detailed items):

*Cultural Adaptation Scale*: Adapted from the Sociocultural Adaptation Scale (SCAS) developed by Ward and Kennedy (1999). This scale has demonstrated good reliability and validity in multinational samples (Cronbach’s *α* > 0.85). The items were slightly modified for the university context, and semantic equivalence for the Chinese and Russian versions was ensured through a back-translation procedure. In this study, Cronbach’s *α* = 0.83.

*Perceived Incentives Scale*: To assess the incentives provided by the university, we adapted items from established scales by Heneman and Schwab (1985) for pay satisfaction and Eisenberger et al. (1986) for perceived organizational support. Conceptually, this scale aims to measure the total package of “organizational value” perceived by the faculty. The scale comprises two dimensions: material incentives (four items, e.g., salary, research funding) and psychological incentives (four items, e.g., academic freedom, recognition of achievement). A Confirmatory Factor Analysis (CFA) showed a good fit for the two-factor model: *χ*^2^*/df* = 2.34, CFI = 0.96, RMSEA = 0.049. In this study, the Cronbach’s *α* was 0.78 for material incentives, 0.79 for psychological incentives, and 0.80 for the total scale.

*Job Satisfaction Scale*: Core items were adopted from the short form of the Minnesota Satisfaction Questionnaire (MSQ-SF) (Weiss et al., 1967), covering intrinsic satisfaction (e.g., sense of achievement, use of abilities) and extrinsic satisfaction (e.g., compensation, working conditions). This scale typically demonstrates a Cronbach’s α between 0.85 and 0.92 globally. In this study, the Cronbach’s α for the total scale was 0.88.

### 3.3 Control variables

To minimize the influence of confounding variables, the following were controlled for in the regression analysis:

*Demographic variables*: Gender (0 = Female, 1 = Male), Age (continuous).

*Professional variables*: Education (1 = Bachelor’s, 2 = Master’s, 3 = PhD), Academic Rank (ordinal), Teaching Tenure (ordinal).

In line with previous expatriate research, these variables are known to be important demographic and professional predictors of job satisfaction and were therefore included as controls. Nationality was treated as a grouping variable for main effect analysis and a moderator for interaction analysis, not as a control variable.

### 3.4 Data analysis strategy

This study used SPSS 26.0 for descriptive statistics, correlation analysis, and reliability tests; AMOS 24.0 for Confirmatory Factor Analysis (CFA); and the PROCESS macro for SPSS (Hayes, 2017) to test for mediation (Model 4) and moderation (Model 1), with the bootstrap resampling set to 5,000 iterations.

## 4 Results

### 4.1 Measurement model assessment

Before hypothesis testing, the data quality was systematically evaluated, including tests for Common Method Bias (CMB), reliability, and validity.

#### 4.1.1 Common method bias (CMB) test

As data were collected via self-report questionnaires, CMB was a potential issue. A combination of procedural controls (e.g., anonymity, item randomization) and statistical tests was employed. Statistically, a CFA model comparison was conducted. The results showed that the theoretical three-factor model (*χ*^2^ = 1235.44, df = 554) had a significantly better fit than a single-factor model where all items were loaded onto one latent variable (*χ*^2^ = 8234.56, df = 560). The chi-square difference was significant (Δ*χ*^2^ = 6999.12, *p* < 0.001), indicating that constructs were empirically distinct and that CMB was not a serious issue.

#### 4.1.2 Reliability and validity test

CFA was used to assess the measurement model. The results indicated that the three-factor model’s fit indices were all within ideal ranges (*χ*^2^/df = 2.23, CFI = 0.94, TLI = 0.93, RMSEA = 0.046), demonstrating a good model-data fit. Specific reliability and validity metrics are shown in Tables 2, 3.

**Table 2:** Reliability and convergent validity of latent variables.

Latent variable	M	SD	Cronbach’s *α*	AVE	CR
Cultural adaptation	3.45	0.60	0.829	0.51	0.85
Perceived incentives	3.58	0.67	0.804	0.54	0.82
Job satisfaction	3.61	0.63	0.877	0.58	0.89

**Table 3:** Discriminant validity test (square root of AVE and correlations).

Variable	1	2	3
1. Cultural adaptation	**0.714**		
2. Perceived incentives	0.41***	**0.735**	
3. Job satisfaction	0.54***	0.62***	**0.762**

Bold diagonal values are the square roots of AVE; *** *p* < 0.001.

### 4.2 Descriptive statistics and national differences

#### 4.2.1 *t*-test for differences between Chinese and Kazakhstani faculty

An independent samples *t*-test was used to analyze differences in core variables between the two national groups (see Table 4). The results showed:

**Table 4:** *t*-test of variables by nationality.

Variable	Nationality	*N*	Mean	SD	*t*	*p*
Cultural adaptation	Chinese	275	3.42	0.58	−1.124	0.262
	Kazakhstani	275	3.48	0.62		
Perceived incentives (total)	Chinese	275	3.51	0.65	−2.451	0.015*
	Kazakhstani	275	3.65	0.68		
Material incentives	Chinese	275	3.44	0.70	−3.102	0.002**
	Kazakhstani	275	3.63	0.72		
Job satisfaction	Chinese	275	3.58	0.61	−0.871	0.384
	Kazakhstani	275	3.63	0.64		

* *p* < 0.05, ** *p* < 0.01.

*No significant difference in job satisfaction and cultural adaptation*: Chinese and Kazakhstani faculty reported similar levels of overall satisfaction (*t* = −0.871, *p* = 0.384) and cultural adaptation (*t* = −1.124, *p* = 0.262).

*Significant difference in perceived incentives*: The mean score for perceived incentives among Kazakhstani faculty (*M = 3.65*) was significantly higher than that of Chinese faculty (*M* = 3.51, *p* = 0.015). This difference was primarily driven by the material incentives dimension (*p* = 0.002), with no significant difference observed in the psychological incentives dimension.

**Figure 1:**
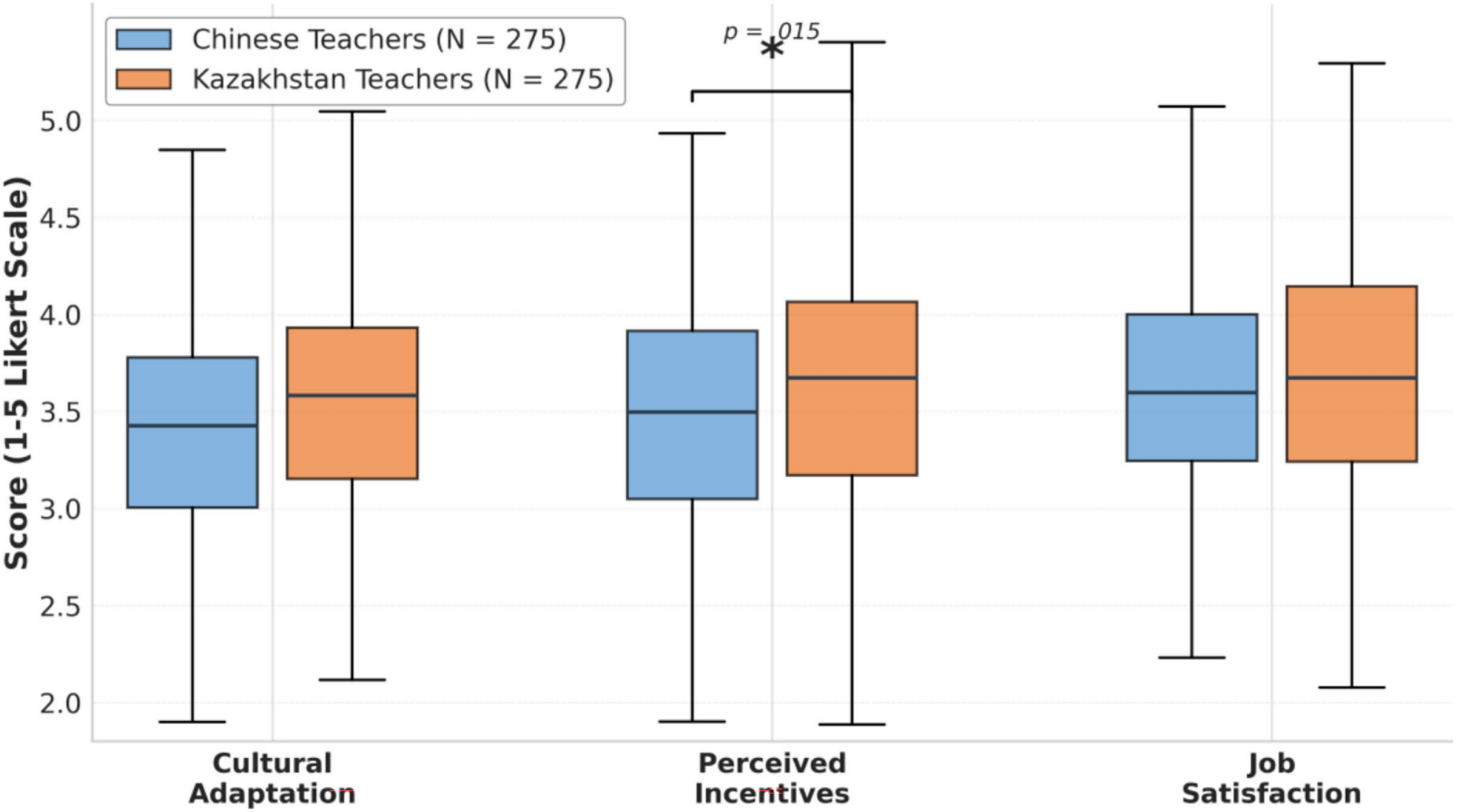
Boxplot comparison of core variables between Chinese and Kazakhstani teachers. Boxes represent interquartile ranges (IQR), horizontal lines indicate medians, and whiskers extend to 1.5 × IQR. The asterisk (*) denotes significant difference (*t* = −2.451, *p* = 0.015) in Perceived Incentives. CA, Cultural Adaptation; PI, Perceived Incentives; JS, Job Satisfaction. Detailed descriptive statistics are presented in Table 4. *N* = 275 per group.

To visually demonstrate these differences, Figure 1 presents the distribution patterns of teachers’ performance across three core variables in both countries through a box-and-whisker plot.

#### 4.2.2 Variable correlation analysis

Table 5 presents the correlation matrix for all variables. Apart from demographic variables, cultural adaptation, perceived incentives, and job satisfaction were all significantly and positively correlated with each other (*r* ranging from 0.41 to 0.62, *p* < 0.001). Control variables (such as academic rank) were also significantly correlated with the dependent variable, providing an empirical basis for their inclusion in the regression analysis.

**Table 5:** Correlation matrix of variables.

Variable	1	2	3	4	5	6	7
1. Gender	1						
2. Age	−0.03	1					
3. Education	0.05	0.18***	1				
4. Academic rank	0.08	0.52***	0.28***	1			
5. Cultural adaptation	−0.02	0.15***	0.09*	0.11*	1		
6. Perceived incentives	0.06	0.09*	0.12**	0.18***	0.41***	1	
7. Job satisfaction	0.04	0.12**	0.14**	0.21***	0.54***	0.62***	1

* *p* < 0.05, ** *p* < 0.01, *** *p* < 0.001.

### 4.3 Hypothesis testing

**Figure 2:**
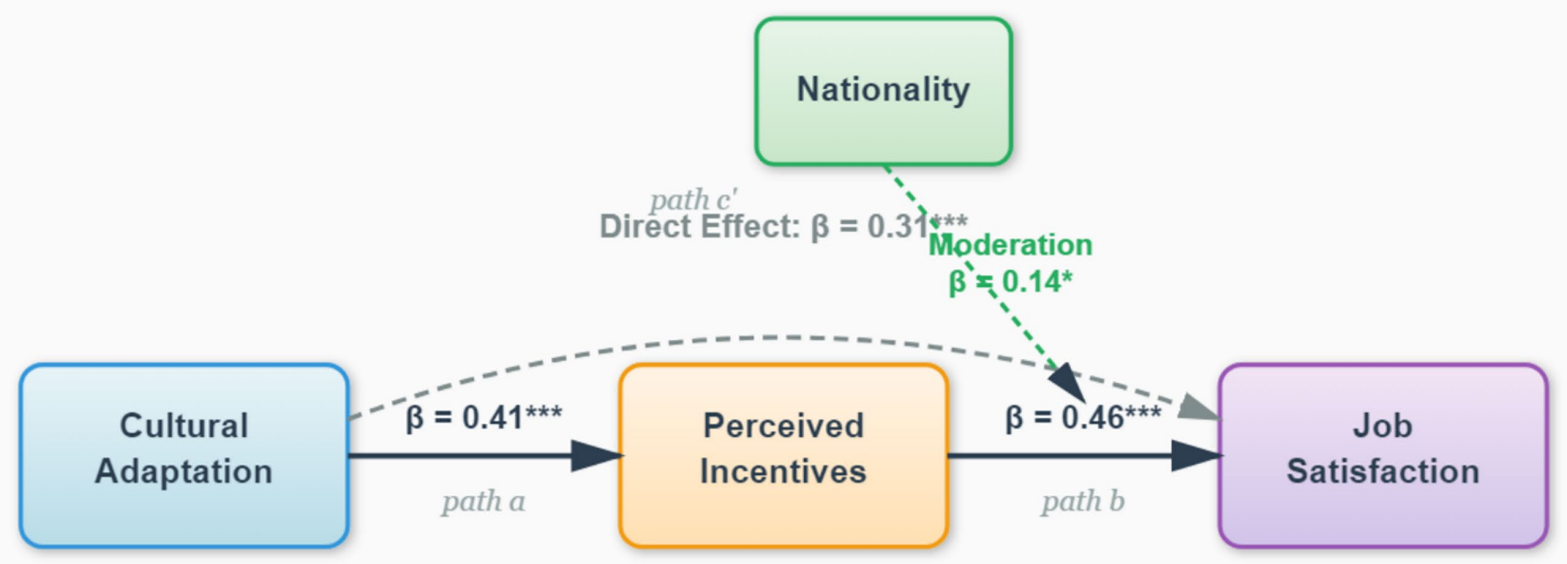
Path model of nationality-moderated mediation effect. Solid arrows represent direct paths; dashed arrows indicate moderation effects. Path coefficients are standardized regression weights derived from Hayes’ PROCESS Model 4 with bootstrap analysis (5,000 resamples, 95% bias-corrected CI). Control variables (gender, age, education, rank, tenure) are included in the analysis but not displayed for clarity. *** *p* < 0.001, ** *p* < 0.01, * *p* < 0.05.

The results of all hypothesis tests are summarized in Figure 2, which displays the complete path model with standardized coefficients and significance levels for the relationships among cultural adaptation, perceived incentives, job satisfaction, and the moderating role of nationality.

#### 4.3.1 Main effects test (H1, H2)

Hierarchical regression analysis was conducted (see Table 6). After controlling for demographic and professional variables (Model 1), cultural adaptation was added (Model 2), showing it significantly and positively predicted job satisfaction (*β* = 0.52, *p* < 0.001). Thus, H1 was supported. In Model 3, perceived incentives were added, also showing a significant positive prediction of job satisfaction (*β* = 0.46, *p* < 0.001). Thus, H2 was supported. The explanatory power of the final model (*R*^2^) reached 48.4%. These results suggest that both adaptation and incentives are crucial independent predictors of satisfaction in this context.

**Table 6:** Hierarchical regression analysis for job satisfaction (*N* = 550).

Predictor	Model 1	Model 2	Model 3
Step 1: control variables			
Gender	0.03	0.01	−0.01
Age	0.07	0.05	0.02
Education	0.09*	0.06	0.03
Academic rank	0.15**	0.11*	0.07
Tenure	0.08*	0.04	0.01
Step 2: independent variable			
Cultural adaptation (CA)		0.52***	0.31***
Step 3: mediator variable			
Perceived incentives (PI)			0.46***
Model statistics			
*F*-value	6.21***	45.89***	83.15***
*R*^2^	0.053	0.321	0.484
Adjusted *R*^2^	0.044	0.314	0.478
Δ*R*^2^	0.053	0.268	0.163
Δ*F*	6.21***	201.75***	158.42***

Values are standardized regression coefficients (*β*); * *p* < 0.05, ** *p* < 0.01, *** *p* < 0.001.

#### 4.3.2 Mediation effect test (H3)

The PROCESS macro (Model 4) was used. The bootstrap results (see Table 7) showed a significant indirect effect of perceived incentives in the relationship between cultural adaptation and job satisfaction. The indirect effect value was 0.232, with a 95% confidence interval of [0.171, 0.298] (not including 0). This indirect effect accounted for 42.7% of the total effect. Since the direct effect also remained significant, this indicates partial mediation. Thus, H3 was supported. This finding implies an associative path where higher adaptation accounts for better perception of incentives, which is then linked to higher satisfaction.

**Table 7:** Bootstrap test for mediation effect.

Path	Effect	BootSE	95% LLCI	95% ULCI
Total effect	0.543	0.041	0.462	0.624
Direct effect	0.311	0.038	0.235	0.386
Indirect effect	0.232	0.032	0.171	0.298

#### 4.3.3 Moderation effect test (H4)

The PROCESS macro (Model 1) was used to test the moderating role of nationality. The results (see Table 8) showed a significant interaction term between perceived incentives and nationality (*β* = 0.14, *p* = 0.020). A simple slope analysis revealed that the sensitivity of Kazakhstani faculty to perceived incentives (Slope = 0.72) was significantly greater than that of Chinese faculty (Slope = 0.58). This means that as perceived incentives increase, the job satisfaction of Kazakhstani faculty is associated with a stronger increase compared to that of Chinese faculty. Thus, H4 was supported.

**Table 8:** Moderation analysis of nationality.

Predictor	Coeff	SE	*t*	*p*	95% CI
Constant	3.61	0.02	158.42	0.000	[3.56, 3.65]
Perceived incentives (X)	0.58	0.04	14.50	0.000	[0.50, 0.66]
Nationality (W)	0.05	0.03	1.67	0.096	[−0.01, 0.11]
Interaction (X × W)	0.14	0.06	2.33	0.020	[0.02, 0.26]

**Figure 3:**
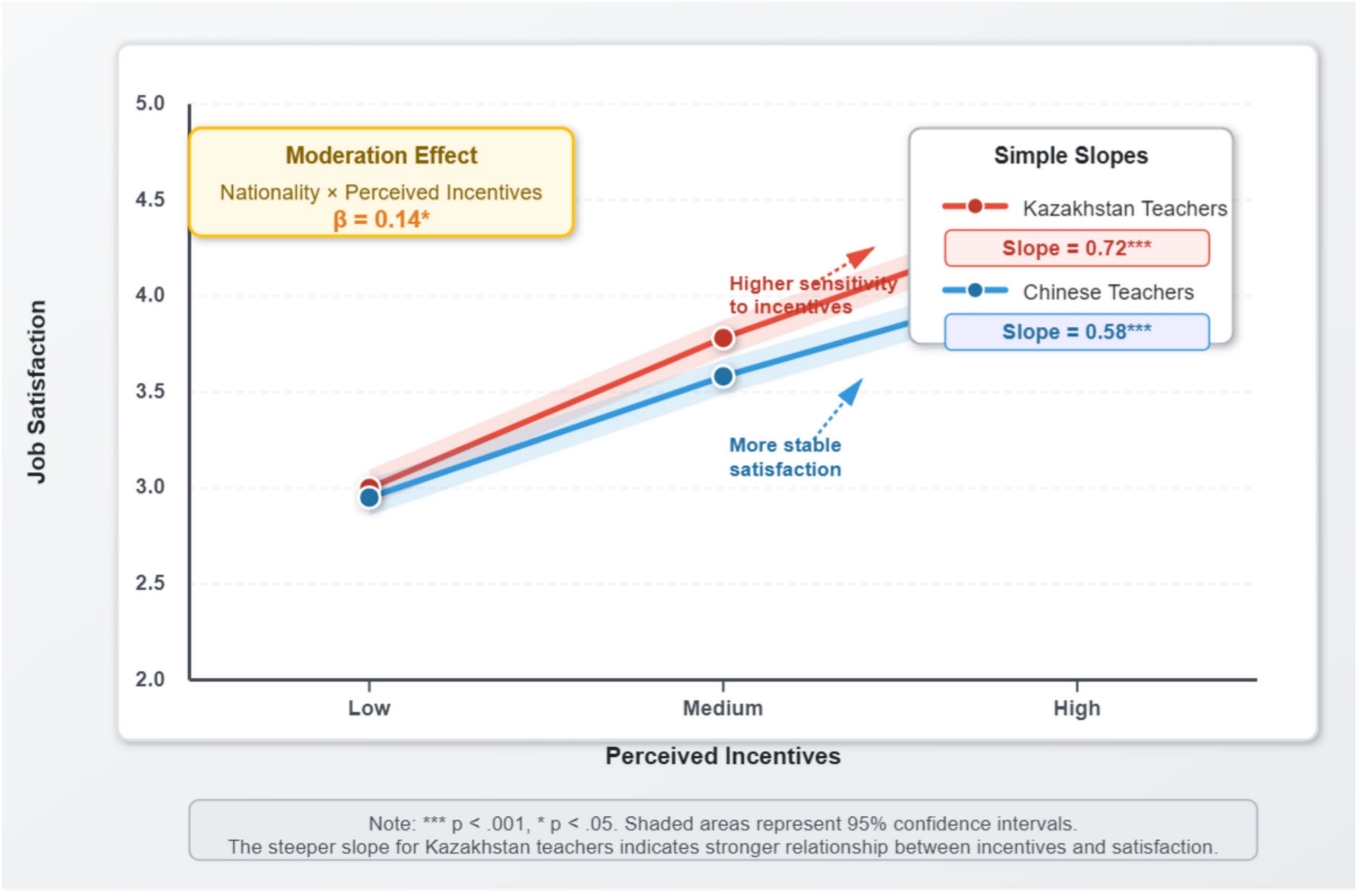
Moderating effect of nationality on the relationship between perceived incentives and job satisfaction. Shaded areas represent 95% confidence intervals. The interaction term (*β* = 0.14, *p* = 0.020) indicates that nationality significantly moderates the incentives-satisfaction relationship. Simple slope analysis and statistical details are displayed within the figure. *** *p* < 0.001, * *p* < 0.05.

To further elucidate the moderating effect of nationality, we conducted a simple slope analysis and plotted an interaction plot, as shown in Figure 3.

### 4.4 Exploratory analysis: the impact of academic rank and tenure on job satisfaction

To address *RQ3*, a two-way ANOVA was conducted to examine the effects of academic rank and teaching tenure on job satisfaction. For this analysis, academic rank was collapsed into three categories (Lecturer/Assistant, Associate Professor, Professor and above), and tenure was collapsed into three categories (<1 year, 1–3 years, >3 years).

The ANOVA results (see Table 9) revealed:

**Table 9:** Two-way ANOVA for job satisfaction by academic rank and teaching tenure.

Source	SS	df	MS	*F*	*p*	Partial *η*^2^
Academic rank	3.89	2	1.94	4.88	0.008	0.018
Tenure	4.95	2	2.47	6.21	0.002	0.022
Rank × tenure	1.74	4	0.43	1.09	0.361	0.008
Error	215.23	541	0.40			
Total	7415.89	550				

SS, sum of squares; df, degrees of freedom; MS, mean square; *η^2^_p_*, partial eta-squared.

A significant main effect for teaching tenure [*F*(2, 541) = 6.21, *p* = 0.002, *η^2^_p_* = 0.022]. A post-hoc test (LSD) showed that the faculty group with 1–3 years of service (M = 3.48, SD = 0.65) had significantly lower job satisfaction than both the group with less than 1 year (M = 3.69, *p* = 0.011) and the group with more than 3 years (M = 3.65, *p* = 0.024). This confirms the existence of an “adaptation slump” or “satisfaction dip” in expatriate teaching careers.

A significant main effect for academic rank [*F*(2, 541) = 4.88, *p* = 0.008, *η^2^_p_* = 0.018]. The post-hoc test showed that the Lecturer/Assistant group (M = 3.51, SD = 0.62) had significantly lower satisfaction than both the Associate Professor group (M = 3.67, *p* = 0.015) and the Professor group (M = 3.73, *p* = 0.005).

The interaction effect between rank and tenure was not significant [*F*(4, 541) = 1.09, *p* = 0.361]. This suggests that the “U-shaped” effect of tenure on satisfaction is consistent across different academic ranks, and the satisfaction differences attributable to rank do not significantly change with increasing tenure.

**Figure 4:**
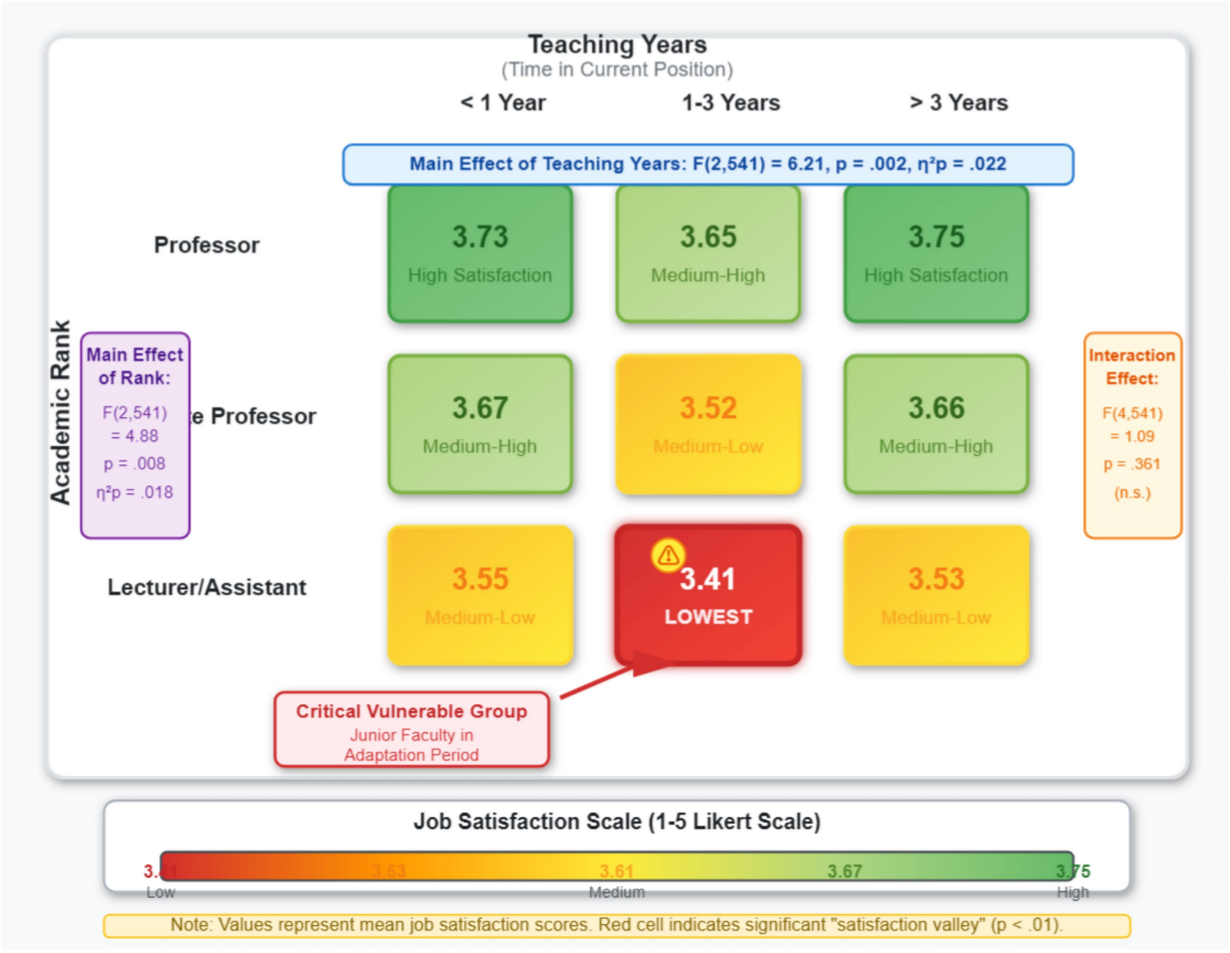
Heatmap of job satisfaction by academic rank and teaching tenure. Data derived from two-way ANOVA (Table 9, *N* = 550). See figure for detailed statistical results.

The results of the two-way ANOVA are presented more intuitively through a heat map, as shown in Figure 4.

**Figure 5:**
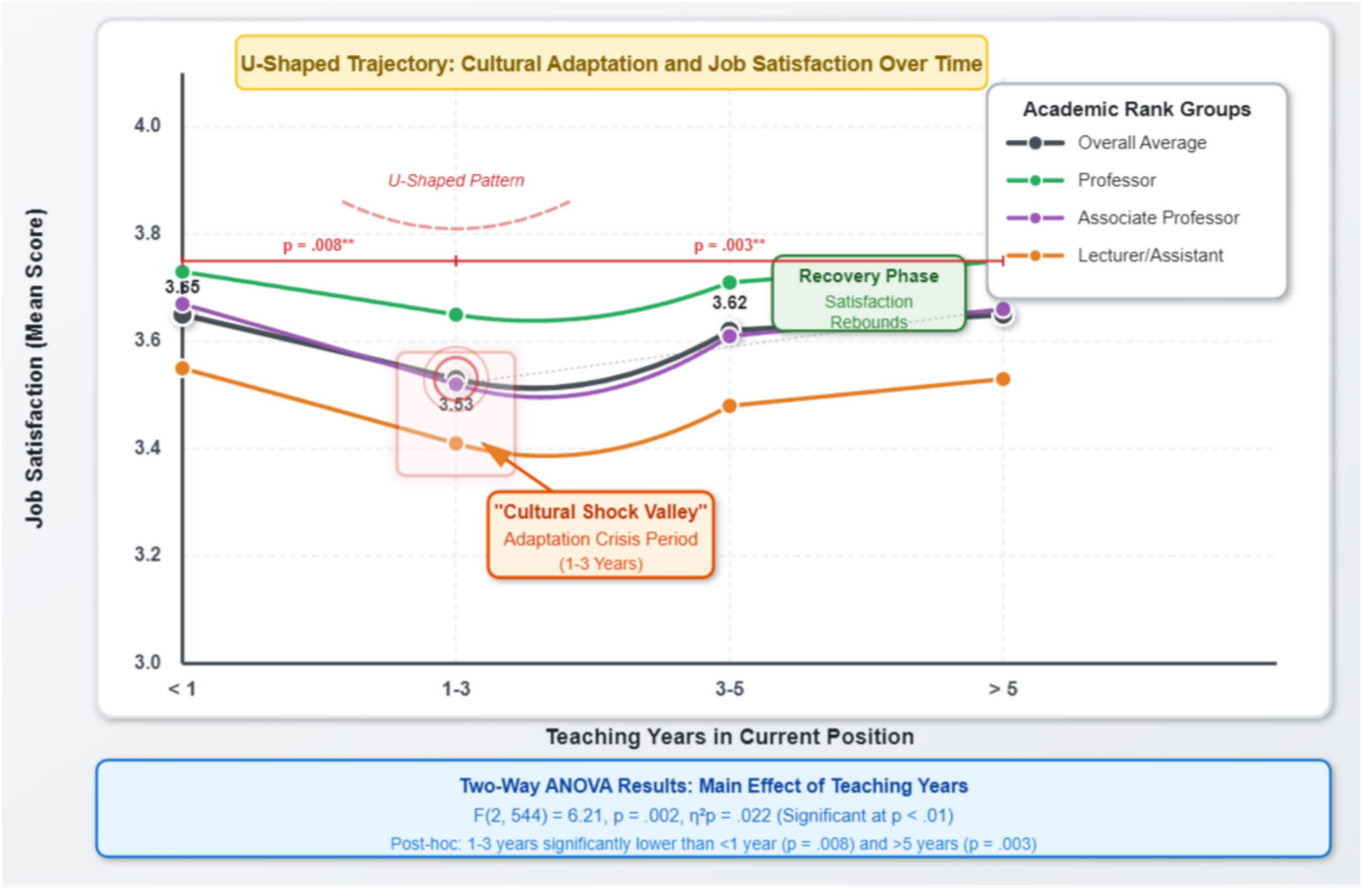
U-shaped trajectory of job satisfaction over teaching tenure by academic rank. Error bars represent 95% confidence intervals. Data derived from two-way ANOVA (Table 9, *N* = 550). The interaction effect between rank and tenure was not significant (*p* = 0.361), indicating the U-shaped pattern is consistent across all academic levels.

The main effect of teaching years on job satisfaction follows a distinct U-shaped curve, as illustrated in Figure 5.

## 5 Discussion

### 5.1 Key findings

Through an empirical survey of 550 Chinese and Kazakhstani expatriate faculty, this study systematically investigated the complex relationships between cultural adaptation, perceived incentives, and job satisfaction. Four core findings emerged:

*Direct effects*: Both cultural adaptation and perceived incentives are significant positive predictors of job satisfaction (supporting H1 and H2), with perceived incentives having a slightly stronger predictive power (*β* = 0.46) than cultural adaptation (β = 0.31, after controlling for the mediator).

*Mediation mechanism*: Perceived incentives play a significant partial mediating role in the relationship between cultural adaptation and job satisfaction (supporting H3), with the indirect effect accounting for 42.7% of the total effect. This reveals a key psychological mechanism through which cultural adaptation influences satisfaction: adaptation is not just a psychological state but also a “resource perception capability.”

*Moderation effect*: Nationality significantly moderates the relationship between perceived incentives and job satisfaction (supporting H4). Kazakhstani faculty are more sensitive to incentive measures than their Chinese counterparts, exhibiting stronger “instrumental rationality.”

*Identification of vulnerable groups*: The two-way ANOVA confirmed the profile of “vulnerable groups” among expatriate faculty. Both teaching tenure and academic rank have significant main effects on job satisfaction, with no interaction between them. Specifically, faculty in their first to third year of service and those with the rank of Lecturer represent clear “satisfaction troughs,” providing a precise target for management interventions.

### 5.2 Theoretical contributions and dialogue

This study aims to bridge the gap between cross-cultural psychology and organizational behavior within the emerging context of “South–South mobility.” By integrating empirical evidence with theoretical dialogue, this research makes three distinct contributions that challenge and extend existing paradigms.

First, this study establishes a “cognitive-instrumental” explanatory pathway for acculturation theory, supplementing the traditional affective perspective. While mainstream acculturation research (e.g., Ward, 2001) predominantly outlines an “adapt well → feel good → be satisfied” emotional pathway, our findings regarding the mediating role of perceived incentives suggest a parallel “adapt → acquire resources → be satisfied” cognitive mechanism. We propose that cultural adaptation possesses significant instrumental value. Adaptation is not merely a state of psychological comfort but may function as a form of capital that is linked to an individual’s capability to decode organizational scripts and acquire scarce resources (e.g., funding, promotion opportunities). This finding shifts the theoretical focus of adaptation from a passive adjustment process to an active resource-acquisition strategy.

Second, this study provides theoretical contextualization for Psychological Contract Theory within the specific realm of “South–South Mobility.” Since transactional and relational contracts were not directly measured, we interpret the observed divergence in incentive sensitivity through this theoretical lens. Specifically, the results for Kazakhstani faculty are consistent with a transactional contract orientation (emphasizing tangible, short-term exchanges), whereas the patterns observed among Chinese faculty align more closely with relational contract expectations (valuing long-term security and institutional mission). This interpretation offers a nuanced framework for understanding expatriate motivation beyond the classic Western-centric models.

Third, this study offers a critical re-examination of the “satisfaction” construct through the lens of Cross-Cultural Equivalence. A counter-intuitive yet profound finding of this research is the lack of a significant quantitative difference in overall job satisfaction between Chinese and Kazakhstani faculty (a “null difference”), despite vast disparities in their institutional environments. We argue that this statistical similarity likely masks a qualitative divergence in the meaning of satisfaction, raising questions about the assumption of construct equivalence. For Chinese faculty embedded in a high-context culture, the self-reporting of “satisfaction” might be moderated by a cultural ethos of content. Conversely, for Kazakhstani faculty, “satisfaction” is likely interpreted more individualistically. Thus, while statistical measurements are similar, the underlying psychological reality may differ.

### 5.3 Managerial implications

Based on our findings, we offer the following specific recommendations for Chinese and Kazakhstani universities to enhance their management of expatriate faculty:

#### Shift from “reactive fixes” to “full-cycle support”

Universities should establish a support system covering the entire cycle: pre-arrival, mid-adaptation, and development phases.

*Pre-arrival*: Provide clear explanations of the compensation structure (especially taxes and insurance) and cross-cultural training to manage expectations and prevent psychological contract breaches due to information asymmetry.

*Mid-adaptation (the 1–3 year crisis period)*: Identify and focus on junior lecturers at the bottom of the “U-shaped curve.” Assign them “dual mentors” (an academic mentor and a life mentor), organize regular feedback sessions, and moderately relax initial research assessment standards to provide an “adaptation buffer.”

*Development phase (>3 years)*: For high-performing faculty, offer more stable, long-term contracts and clear career advancement paths to transition them from “transient visitors” into “vested stakeholders.”

#### Move from a “one-size-fits-all” to a “precision incentive” approach

Given the significant moderating effect of nationality, universities should abandon a monolithic incentive model and implement a differentiated “menu of incentives.”

*For Kazakhstani faculty (transactional)*: The focus should be on material and performance-based incentives. Establish a transparent and competitive salary system, closely link research output to performance bonuses, and provide ample start-up research funding. Streamlining administrative and financial reimbursement processes is key to boosting their satisfaction.

*For Chinese faculty (relational)*: The focus should be on psychological and belongingness needs. As Murali et al. (2025) suggest, unpacking the cultural factors that shape adaptation and belonging is key. The sending institution in China should reinforce their sense of mission and organizational identity. Furthermore, host universities should actively facilitate the building of robust social networks to prevent expatriates from experiencing the “fragile lives” that result from “weak ties” (Ma and Black, 2025), offsetting any shortfalls in material conditions abroad.

#### Transition from “passive management” to “proactive early warning”

Universities should create a dynamic monitoring and early-warning system for expatriate faculty satisfaction. For example, conduct a brief, anonymous survey each semester, focusing on the lecturer group in the “1–3 year crisis period.” If satisfaction levels fall below a warning threshold, the human resources department or international office should proactively intervene to understand the specific difficulties and provide personalized solutions, nipping turnover risks in the bud.

Finally, beyond institutional policy, this study offers actionable takeaways for individual expatriate faculty to enhance their own cross-cultural journey:

*Active resource seeking (cognitive strategy)*: Faculty should recognize that cultural adaptation is an instrumental tool for resource acquisition. Instead of passively waiting for support, they should actively study the host university’s “cultural dynamics” (e.g., funding mechanisms, unwritten promotion rules) and proactively utilize available incentives to fuel their professional growth.

*Navigating the “1-to-3 year dip”*: It is crucial for faculty to realize that the satisfaction slump in the first 3 years is a common career stage, not a personal failure. During this period, actively seeking “social support”—such as joining local academic communities or finding a mentor—is more effective than isolation.

*Managing psychological contracts*: Faculty are advised to clarify their expectations early on. Understanding whether their motivation is primarily transactional (financial gain) or relational (mission-driven) can help them better align their personal goals with the organization’s reality, thereby reducing psychological gaps.

### 5.4 Limitations and future research

This study has several limitations that also point to directions for future research:

*Cross-sectional design*: The use of cross-sectional data limits our ability to make causal inferences. The relationships identified as “predicting” or “enhancing” should be understood as associational. Future research should employ longitudinal designs to better establish temporal ordering.

*Measurement equivalence*: While we compared means and tested moderation, strict measurement invariance testing across national groups was not the primary focus of this study. Therefore, findings related to national differences should be interpreted with caution. Future research should conduct rigorous multi-group analysis (MGCFA) to establish full metric and scalar invariance.

*Common method bias*: Despite procedural and statistical controls, self-report questionnaires may still be subject to social desirability bias. Future studies could incorporate multi-source data (e.g., peer or dean evaluations) or objective metrics (e.g., turnover rates, publication counts).

*Sample representativeness*: The sample was primarily drawn from universities in central and western China and had an uneven distribution across disciplines. Future research could broaden the sample scope and compare differences across disciplines (e.g., humanities vs. STEM).

*Variable limitations*: This model did not include other important variables such as family factors (spousal adaptation, children’s education) or personality traits (the Big Five). Future research could build more comprehensive, integrated models.

## 6 Conclusion

### 6.1 Research conclusions

This study systematically examined the relationships among cultural adaptation, perceived incentives, and job satisfaction for expatriate faculty exchanged between China and Kazakhstan, leading to the following core conclusions:

First, cultural adaptation is a crucial foundation for job satisfaction, and its effect is partially realized by its association with enhancing the perception of incentives. Well-adapted faculty are more likely to perceive organizational incentives and support, thereby achieving higher satisfaction. This reveals the key mediating chain of “Adaptation → Perceived Incentives → Satisfaction.”

Second, the effectiveness of incentive measures appears to be culturally contingent. Kazakhstani faculty are more sensitive to material incentives, consistent with a transactional psychological contract, while the satisfaction of Chinese faculty is less affected by fluctuations in external incentives, consistent with the theoretical profile of a relational psychological contract. This demonstrates that a “one-size-fits-all” incentive policy is likely to be suboptimal.

Third, this study scientifically identified “vulnerable groups” in expatriate faculty management through variance analysis. Regardless of nationality or cultural background, faculty in their first to third year of service and those with the rank of Lecturer both reported significantly lower job satisfaction. This indicates the existence of a universal “adaptation bottleneck” and a “rank-related pressure period” in expatriate academic careers, representing the phase with the highest turnover risk and the greatest need for organizational support.

### 6.2 Concluding remarks

In an era where globalization and de-globalization forces intertwine, expatriate university faculty serve as envoys of knowledge and bridges for cultural exchange. Their professional well-being is not just a personal matter but is integral to the depth and sustainability of educational cooperation between nations. The findings of this study demonstrate that cultural adaptation is not a spontaneous, natural process, nor do incentives follow a simple logic of “just pay them enough.” Only by deeply understanding the psychological needs of faculty from different cultural backgrounds, precisely identifying the pain points at various career stages, and systematically building a full-cycle support system can the strategic goal to “attract, retain, and effectively utilize talent” be truly achieved. As the Kazakh poet Abai Qunanbaiuly said, “Knowledge is like a light that illuminates the path forward.” May the educational envoys of China and Kazakhstan, on their cross-cultural journey, not only adapt to the foreign land but also feel the warmth of their organizations, ultimately reaping professional fulfillment and life meaning.

### 6.3 Ethics approval and consent to participate

This study was conducted in strict compliance with the ethical principles of the Declaration of Helsinki and China’s relevant ethical policies for academic research (including the exemption provisions for non-invasive and anonymous social science surveys in the Measures for the Ethical Review of Biomedical Research Involving Humans). As a non-invasive and anonymous educational survey that does not involve sensitive or private content, formal approval from an Institutional Review Board (IRB) was not required in accordance with the academic practices of the applicant’s affiliated institution—Continuing Education College, Chang’an University—and the requirements of China’s relevant policies.

All participants volunteered to participate in the study. Prior to completing the questionnaire, they were required to review a bilingual (Chinese/Russian) informed consent statement, which clearly outlined the study’s purpose, data usage, and their rights (including the right to withdraw at any time without adverse consequences). Participants could only access the questionnaire after checking the consent box. Research data were collected and stored anonymously, used exclusively for academic analysis, and strictly complied with China’s relevant regulations on data privacy protection to effectively safeguard participants’ privacy.

## Data Availability

The original contributions presented in the study are included in the article/Supplementary material, further inquiries can be directed to the corresponding author.
