# Supplementary material for: Cultural adaptation, perceived incentives, and job satisfaction of expatriate faculty: an empirical study of China and Kazakhstan
Source: Front Psychol. 2026 Feb 17;17:1781942. doi: 10.3389/fpsyg.2026.1781942 (PMC12953139; doi:10.3389/fpsyg.2026.1781942)
Supplement: Supplementary file 1 [file Data_Sheet_1.zip › Supplementary Material A Survey Instrument (Bilingual Version).docx]

**Supplementary Material A: Survey Instrument (Bilingual Version)**
**附录材料 A：调查工具**

**TITLE: Study on the Correlation between Acculturation, Perceived Incentives, and Job Satisfaction of University Teachers in China and Kazakhstan**
**题目：中哈大学教师文化适应、激励感知与职业满意度的关联研究**

### Informed Consent / 知情同意 /  Информированное согласие

**致参与者 / Участникам:**
本研究旨在探讨中哈高校教师的跨文化适应与职业发展。研究遵循匿名、保密和自愿原则。

1. **匿名性：** 问卷不收集姓名、身份证号等个人识别信息。
2. **保密性：** 数据仅供学术研究团队分析使用。
3. **自愿性：** 您有权在任何时候中止填答，这不会对您的工作产生任何影响。
   完成本问卷约需 10-15 分钟。
   Данное исследование направлено на изучение межкультурной адаптации и профессионального развития преподавателей университетов в Китае и Казахстане. Исследование основано на принципах анонимности, конфиденциальности и добровольности.
4. **Анонимность:** Анкета не собирает личные данные (имя, номер ID и т.д.).
5. **Конфиденциальность:** Данные будут использоваться только исследовательской группой в научных целях.
6. **Добровольность:** Вы имеете право прекратить участие в любой момент, это никак не повлияет на вашу работу.
   Заполнение анкеты займет около 10-15 минут.

**[ ] 我已阅读上述说明，并同意参与本项调查。**
**[ ] Я прочитал(-а) вышеизложенное и согласен(-на) участвовать в исследовании.**

### **Section I: Demographic Information (Основные сведения)**

**1. 国籍 / Гражданство:**
□ 中国 / Китай
□ 哈萨克斯坦 / Казахстан
□ 其他 / Другое

**2. 性别 / Пол:**
□ 男 / Мужской
□ 女 / Женский

**3. 年龄 / Возраст:**
__________ 岁 / лет

**4. 最高学历 / Уровень образования:**
□ 学士 / Бакалавр
□ 硕士 / Магистр
□ 博士 / Доктор наук (PhD)

**5. 在东道国居住/工作时间 / Срок проживания/работы в принимающей стране:**
□ 1年以下 / Менее 1 года
□ 1-3年 / 1-3 года
□ 3-5年 / 3-5 лет
□ 5年以上 / Более 5 лет

**6. 东道国语言熟练度 / Владение языком принимающей страны:**
□ 完全不会 / Не владею
□ 略懂 / Слабо владею
□ 基本交流 / Базовый уровень
□ 流利 / Свободно владею
□ 精通 / Профессиональный уровень

**7. 职称 / Академическое звание:**
□ 助教/讲师 / Ассистент/Преподаватель
□ 副教授 / Ассоциированный профессор (Доцент)
□ 教授 / Профессор
□ 其他 / Другое

### **Section II: Acculturation Scale (Шкала социокультурной адаптации)**

*(Source: SCAS; Ward & Kennedy, 1999)*

**指导语：** 请评估您在当前国家生活和工作中处理以下情况的困难程度。
**Инструкция:** Оцените уровень трудности, с которым вы сталкиваетесь в следующих ситуациях в принимающей стране.
*(1 = Нет трудностей / 没有困难; 5 = Очень трудно / 非常困难)*

| No. | Items (题目 / Вопросы) |
| --- | --- |
| 8 | 结交当地朋友 Заводить друзей среди местных жителей |
| 9 | 使用当地的交通系统 Пользоваться местной транспортной системой |
| 10 | 适应当地的饮食习惯 Привыкать к местной еде |
| 11 | 遵循当地的社会规范和规则 Соблюдать местные социальные нормы и правила |
| 12 | 处理行政事务或官僚程序 Решать административные вопросы или бюрократические процедуры |
| 13 | 理解当地人的幽默或玩笑 Понимать местный юмор и шутки |
| 14 | 在工作中与当地同事沟通 Общаться с местными коллегами по работе |
| 15 | 适应当地学校的管理风格 Адаптироваться к стилю управления в местном университете |
| 16 | 处理因文化差异引起的误解 Справляться с недопониманием из-за культурных различий |
| 17 | 作为一个外国人居住在这里的感觉 Ощущать себя иностранцем, проживающим здесь |

### **Section III: Perceived Incentives Scale (Шкала восприятия стимулов)**

*(Adapted from Heneman & Schwab, 1985; Eisenberger et al., 1986)*

**指导语：** 请评估您对所在高校提供的下列支持/激励措施的同意程度。
**Инструкция:** Оцените степень вашего согласия со следующими утверждениями о поддержке/стимулах в вашем вузе.
*(1 = Полностью не согласен / 非常不同意; 5 = Полностью согласен / 非常同意)*

| No. | Items (题目 / Вопросы) |
| --- | --- |
| 18 | 我目前的薪资水平与我的工作量是匹配的。 Мой уровень зарплаты соответствует моей рабочей нагрузке. |
| 19 | 学校提供的住房补贴或住宿条件令我满意。 Я удовлетворен(-а) жилищной субсидией или условиями проживания, предоставляемыми вузом. |
| 20 | 学校的科研启动经费和教学设施支持充足。 Финансирование научных исследований и учебная база вуза являются достаточными. |
| 21 | 相比于我的祖国，目前的福利待遇更具吸引力。 По сравнению с моей родной страной, нынешний социальный пакет более привлекателен. |
| AC1 | (质量检测题) 为了确保数据质量，请本题直接选择“2”。 (Контроль качества) Для проверки внимания, пожалуйста, выберите вариант “2”. |
| 22 | 学校有着公平的职称晋升和职业发展通道。 В вузе существуют справедливые каналы для продвижения по службе и профессионального развития. |
| 23 | 我的教学和科研成果得到了领导和同事的认可。 Мои достижения в преподавании и науке признаются руководством и коллегами. |
| 24 | 学校经常提供跨文化交流培训或学术进修机会。 Вуз часто предоставляет возможности для межкультурного тренинга или академической стажировки. |
| 25 | 我在这里感受到了充分的学术自由和尊重。 Здесь я чувствую полную академическую свободу и уважение. |

### **Section IV: Job Satisfaction Scale (Шкала удовлетворенности работой)**

*(Source: MSQ-Short Form; Weiss et al., 1967)*

**指导语：** 请问您对当前工作的下列各个方面是否感到满意？
**Инструкция:** Насколько вы удовлетворены следующими аспектами вашей нынешней работы?
*(1 = Крайне не удовлетворен / 极不满意; 5 = Полностью удовлетворен / 极满意)*

| No. | Items (题目 / Вопросы) |
| --- | --- |
| 26 | 能够一直保持忙碌的工作状态。 Возможность быть постоянно занятым работой. |
| 27 | 能够独立处理工作。 Возможность работать самостоятельно. |
| 28 | 能够做些不同的事情（工作多样性）。 Возможность делать разные вещи (разнообразие в работе). |
| 29 | 我在社会上的地位。 Положение, которое я занимаю в обществе благодаря работе. |
| 30 | 领导处理下属关系的方式。 То, как мой руководитель обращается с подчиненными. |
| 31 | 领导决策的能力。 Компетентность моего руководителя в принятии решений. |
| 32 | 能够做一些不违背良心的事情。 Возможность делать то, что не противоречит моей совести. |
| 33 | 工作的稳定性。 Стабильность моей работы. |
| 34 | 能够为他人做些事情（社会服务）。 Возможность делать что-то для других людей. |
| 35 | 有指导别人的机会。 Возможность давать указания другим людям. |
| 36 | 利用自己能力的机会。 Возможность использовать свои способности. |
| 37 | 学校制度及其实施的方式。 Политика вуза и то, как она реализуется. |
| 38 | 我的收入与工作量相比（薪酬）。 Моя зарплата по сравнению с объемом работы. |
| 39 | 职位的晋升机会。 Возможности для продвижения по службе. |
| 40 | 对工作自由做出判断的自由（责任感）。 Свобода использовать собственное суждение в работе. |
| 41 | 工作本身带来的成就感。 Чувство достижения, которое я получаю от работы. |
| 42 | 总体来说，我对目前这份工作感到满意。 В целом, я удовлетворен(-а) своей нынешней работой. |

**References for the Survey Instruments:**

Eisenberger, R., Huntington, R., Hutchison, S., & Sowa, D. (1986). Perceived organizational support. Journal of Applied Psychology, 71(3), 500–507.

Heneman, H. G., & Schwab, D. P. (1985). Pay satisfaction: Its multidimensional nature and measurement. International Journal of Psychology, 20(2), 129–141.

Weiss, D. J., Dawis, R. V., England, G. W., & Lofquist, L. H. (1967). Manual for the Minnesota Satisfaction Questionnaire. Minnesota Studies in Vocational Rehabilitation, 22, 120.

Ward, C., & Kennedy, A. (1999). The measurement of sociocultural adaptation. International journal of intercultural relations, 23(4), 659-677.<https://doi.org/10.1016/S0147-1767(99)00014-0>
